# Evaluation of islets derived from human fetal pancreatic progenitor cells in diabetes treatment

**DOI:** 10.1186/scrt352

**Published:** 2013-11-22

**Authors:** Wen-Jian Zhang, Shi-Qing Xu, Han-Qing Cai, Xiu-Li Men, Zai Wang, Hua Lin, Li Chen, Yong-Wei Jiang, Hong-Lin Liu, Cheng-Hui Li, Wei-Guo Sui, Hong-Kui Deng, Jin-Ning Lou

**Affiliations:** 1 Institute of Clinical Medical Sciences, China-Japan Friendship Hospital, Beijing 100029, P. R. China; 2 Department of Endocrinology, Second Hospital of Jilin University, Changchun 130041, Jilin, P. R. China; 3 Department of Pathophysiology, Hebei United University, Tangshan 063000, China; 4 Department of Gynecology & Obstetrics, China-Japan Friendship Hospital, Beijing 100029, P. R. China; 5 Department of Endocrinology, Qilu Hospital, Shandong University, Jinan 250012, Shandong, China; 6 First Kidney Transplantation Hemopurification Center of Chinese PLA, 181st Hospital of Guangzhou Military Area of PLA, Guilin, China; 7 Key Laboratory of Cell Proliferation & Differentiation of Ministry of Education, College of Life Sciences, Peking University, Beijing 100871, China

## Abstract

**Introduction:**

With the shortage of donor organs for islet transplantation, insulin-producing cells have been generated from different types of stem cell. Human fetal pancreatic stem cells have a better self-renewal capacity than adult stem cells and can readily differentiate into pancreatic endocrine cells, making them a potential source for islets in diabetes treatment. In the present study, the functions of pancreatic islets derived from human fetal pancreatic progenitor cells were evaluated *in vitro* and *in vivo*.

**Methods:**

Human pancreatic progenitor cells isolated from the fetal pancreas were expanded and differentiated into islet endocrine cells in culture. Markers for endocrine and exocrine functions as well as those for alpha and beta cells were analyzed by immunofluorescent staining and enzyme-linked immunosorbent assay (ELISA). To evaluate the functions of these islets *in vivo*, the islet-like structures were transplanted into renal capsules of diabetic nude mice. Immunohistochemical staining for human C-peptide and human mitochondrion antigen was applied to confirm the human origin and the survival of grafted islets.

**Results:**

Human fetal pancreatic progenitor cells were able to expand in medium containing basic fibroblast growth factor (bFGF) and leukemia inhibitor factor (LIF), and to differentiate into pancreatic endocrine cells with high efficiency upon the actions of glucagon-like peptide-1 and activin-A. The differentiated cells expressed insulin, glucagon, glucose transporter-1 (GLUT1), GLUT2 and voltage-dependent calcium channel (VDCC), and were able to aggregate into islet-like structures containing alpha and beta cells upon suspension. These structures expressed and released a higher level of insulin than adhesion cultured cells, and helped to maintain normoglycemia in diabetic nude mice after transplantation.

**Conclusions:**

Human fetal pancreatic progenitor cells have good capacity for generating insulin producing cells and provide a promising potential source for diabetes treatment.

## Introduction

The success of islet transplantation over the last decade suggests that diabetes can be cured by a replenishment of deficient beta cells [1,2]. However, according to the transplantation protocol of Edmonton, the transplanted islets into each patient should be isolated from two to three pancreatic donors, exceeding ten thousand islet equivalent (IEQ)/kg [3]. As a result, a widespread use of islet transplantation is severely limited by the shortage of donor organs. Stem cells have the capacity of self-renewal and the potential of differentiating into various cell types, making them an ideal candidate to address this issue.

Although the possibility of generating insulin-producing cells routinely from both human embryo stem cells (ESC) and human induced pluripotent stem (iPS) cells is quite encouraging, significant challenges still remain [4]. Lumelsky [5] and Assady [6] found that islet-like cells could be generated *in vitro* by differentiation of ESC under conditions resembling those of physiological development for pancreatic beta cell development. However, insulin secretion by these cells was low and lacked a full response to glucose. Clinically, applying ESC-derived cells in treatment presents more challenges including the risk of cancer formation, functional deficiency of such cells and controversial ethical issues. Deriving insulin-releasing cells from iPS cells poses similar problems. Although several small molecules were able to efficiently induce iPS cells into insulin-producing cells, only about 10% of the cells became productive [7].

Human adult stem cells derived from various tissues were also explored for generating insulin-producing cells. Kadam *et al*. expanded mesenchymal stromal cells from human umbilical cord and placenta, and differentiated them into functional islets *in vitro*[8]. Chandra *et al*. [9] reported that islet-like cell aggregates derived from stem cells in human adipose tissue ameliorated experimental diabetes in mice. However, the extent of endocrine cell formation and insulin secretory function were insufficient to become clinically applicable [10].

It has been demonstrated that stem cells are available in pancreatic ducts and islets, with the capability of differentiating into pancreatic exocrine and endocrine cells [11,12], and the number of pancreatic stem cells increased upon partial pancreatectomy or destructive immune response [13]. Thus, it is possible that pancreatic stem cells might be promoted to differentiate into functional islet endocrine cells under *in vitro* culture conditions [14].

It is known that pancreatic stem cells differentiating toward endocrine cells express pancreatic duodenal homeobox-1(PDX-1) and neurogenin 3. Bonner-Weir *et al*. [15] showed that human pancreatic duct cells could be expanded and differentiated into glucose responsive islet tissue *in vitro* given ITS (insulin, transferrin, selenium), nicotinamide and keratinocyte growth factor. Ramiya *et al*. [14] isolated murine pancreatic ductal epithelial cells into culture, and induced them into functional islets containing alpha, beta and delta cells. These resulting islets showed temporal changes in mRNA transcripts for islet cell-associated differentiation markers, responded to glucose challenge *in vitro* and reversed insulin-dependent diabetes after being implanted into non-obese diabetic mice.

While pancreatic stem cells isolated from adult pancreas have low proliferative capability [16], fetal pancreatic cells have shown stronger proliferative potential *in vitro*, even those obtained during the second or third trimester [17]. Moreover, human fetal pancreatic progenitor cells also have the capability of differentiating into insulin-producing cells *in vitro*[18-20]. In the present study, we demonstrate that pancreatic stem cells isolated from human fetal pancreas have the capacity not only to expand and differentiate extensively into islet endocrine cell *in vitro* but also to correct high blood glucose efficiently in diabetic animals.

## Methods

### Isolation, purification and identification of human pancreatic progenitor cells

The present study was approved by the Clinical Research Ethics Committee of China-Japan Friendship Hospital and conducted according to the principles of the Declaration of Helsinki. Five human fetal pancreases at the 10th to 12th gestational week were obtained from abortion patients in China-Japan Friendship Hospital, in which one was a spontaneous abortion due to low progesterone level and the other four were intended abortions according to the mothers’ choice. All the tissues were obtained following medical ethics and all with patient informed consent.

Pancreas tissues at the 10th to 12th gestational week were confirmed to be abundant with islet-like structures which were CD133 positive but insulin negative by immunohistochemistry staining. The pancreatic tissues were digested with XI collagenase (Sigma, Shanghai, China), and the islet-like structures extracted were suspended in (D)MEM/F12 (Sigma) in a 35-mm cell culture dish. After slowly hand-shaking the dish, the islet-like structures would move to the middle of the dish and were picked up using a pipette under a stereomicroscope (Nikon, Beijing, China). The islet-like structures were resuspended and cultured in a 37°C, 5% CO_2_ incubator in (D)MEM/F12 medium containing 5% fetal calf serum for stem cell, 40 μg/L leukemia inhibitor factor (LIF), 10 μg/L basic fibroblast growth factor (bFGF), 10 μg/L epidermal growth factor (EGF), 10^5^ U/L penicillin and 100 mg/L streptomycin [5] Adherent cells that grew from the islet-like structures after 24 hours were trypsinized for passage with 0.1% trypsin/0.1% ethylenediaminetetraacetic acid (EDTA) solution at confluence. The propagated cells were saved for further study. The control human islets were isolated from a section of pancreas after pancreatectomy from a patient with a pancreatic tumor, as previously described [21].

RT-PCR was employed to detect the following markers for proliferated stem cells: Oct4, ATP-binding cassette superfamily G member 2 (ABCG2), stem cell factor (SCF), CD133, carbonic anhydrase II (CAII), cytokeratin 19 (CK19), PDX-1 and neurogenin 3. The expression of PDX-1 and Neurogenin 3 (Ngn3) was also confirmed by immunofluorescence staining using goat anti-human PDX-1 antibody (Abcam, Cambridge, MA, USA) and rabbit anti-human Ngn3 antibody (Abcam). After two, five and ten passages, cells were collected to measure the expression levels of smooth muscle actin (SMA), vimentin, stem cell markers (Oct4, PDX-1 and CA II) and mature cell markers (insulin and glucagon) by real-time PCR.

### Induced differentiation of human pancreatic progenitor cells

Human fetal pancreatic progenitor cells were induced in M199 medium containing 15% fetal bovine serum (FBS), 10 mmol/L nicotinamide, 30 ng/ml all-trans retinoic acid and 42 ng/ml glucagon-like peptide-1 (gift of Shanghai Huayi Bio-Lab Co. Ltd) for four weeks. The medium was replaced every three days and 50 ng/ml activin A was added to the medium in the last week. The flowchart of the differentiation protocol is as follow (Nico, nicotinamide; RA, all-trans retinoic acid):

### Formation of islet-like structures

After four weeks of directed differentiation, the cells were harvested and aggregated in suspension culture under special conditions. In brief, the cells were resuspended in M199 containing 20% FBS, 1.5 mmol/L Ca^2+^, 1 mmol/L ATP, 2 mmol/L glutamine, 2 μg/ml laminin, 5 μg/ml Type IV collagen and 3 μg/ml fibronectin. The mixture was transferred to a nontreated flask or siliconized centrifugal tubes and placed into a 37°C, 5% CO_2_ incubator. Gentle agitation was applied every eight hours. After a 24-hour incubation, small cell aggregations formed and were harvested by centrifugation at 1,200 rpm for eight minutes. The morphology of islet-like structures was examined under a stereomicroscope.

### Immunofluorescent staining

The islets differentiated from progenitor cells were selected manually, embedded in optimal cutting temperature (OCT) compound and prepared into frozen sections. The expressions of insulin and glucagon were examined and compared with adult islets as control. The frozen sections were then fixed for one minute with alcohol (95%), washed three times with PBS solution and blocked for 30 minutes at room temperature with 0.1% BSA/PBS. Then anti-insulin and anti-glucagon antibodies (Sigma) were incubated overnight, respectively, with the cells at 4°C. After the sections were rinsed three times with PBS solution, Alex488-labeled secondary antibody (Invitrogen Inc, Grand Island, NK, USA) was added and incubated with the cells for another 30 minutes. A fluorescent microscope was used to observe the results.

### Transmission electron microscopy

Adult islets and differentiated cells were collected by centrifugation and washed with PBS, then fixed with 2.5% glutaraldehyde for 30 minutes at room temperature. Cells were washed, post-fixed, dehydrated and embedded with Epon 812 (Electron Microscopy Sciences, Beijing, China). The sections were examined under a JEM-1010 electron microscope (JEOL Inc, Tokyo, Japan). Five fields were examined for each sample.

### Real-time PCR

Total RNA was extracted from cells 0, 3 and 4 weeks post differentiation and from islet-like structures 4 weeks post differentiation using a RNEasy Mini kit (Qiagen Inc, Hilden, Germany). A total of 1 μg RNA was used for reverse transcription using omniscript reverse transcriptase (Qiagen). Real-time PCR was performed on an Applied Biosystems instrument (ABI 7500 system), using SYBR Green PCR Master Mix (ABI Inc, Foster city, CA, USA) and 40 reaction cycles. Markers for pancreatic stem cells, endocrine, exocrine and mesenchymal cells were measured at different time points of expansion and differentiation At the end of each reaction, melting-curve analysis was performed to confirm the absence of primer dimers. The expression levels of genes of interest were normalized to the expression level of GAPDH. Data were analyzed using the 2^- Δ Ct^ method. The pancreatic stem cell markers (CAII, PDX-1, Neurogenin 3; endocrine markers: insulin, glucagon, GLUT1, GLUT2 and VDCC), as well as exocrine markers (HES-1 and amylase) were evaluated during differentiation. The mesenchymal markers SMA and vimentin were evaluated during pancreatic stem cell expansion. The gene-specific primer sequences are shown in additional file (see Additional file 1).

### Insulin release assay by ELISA

After pre-incubation with Krebs-Ringer buffer at 37°C for 90 minutes, the progenitor cell-derived islets (100 IEQs/ml) were incubated with Krebs-Ringer buffer containing 2.5 mM glucose or 25 mM glucose at 37°C for different durations. Conditioned medium was then collected, centrifuged and assayed for insulin content by an ELISA kit (Linco Inc, St Charles, MO, USA).

### Animal experiments

#### ***Diabetic animal models***

The animal study was approved by the Animal Ethics Committee of China-Japan Friendship Hospital. Twelve male nude BALB/c mice, four- to six-weeks-old, were purchased from Vital River Laboratories (quality certificate: SCXK (Beijing) 20060009)–. All animals were housed in the specific pathogen free (SPF) facility of the Institute of Clinical Research at our hospital. The diabetic model was induced in the mice by an intraperitoneal injection of streptozocin (200 mg/kg body weight; Sigma, St. Louis, MO, USA) freshly dissolved in citrate buffer (pH 4.5). Blood samples were taken from the tail vein of the animals under non-fasting conditions (8:00 A.M.; standard laboratory diet *ad libitum* overnight) and examined for blood sugar level using a glucometer (Onetouch®Ultra Easy™, Johnson & Johnson, Shanghai, China). Only animals exhibiting blood glucose concentrations >20 mM in three consecutive measurements were employed in the study. They were assigned randomly into a control group and a progenitor cell-derived islet group (n = 6 each).

#### ***Transplantation of differentiated islet-like structures***

To evaluate the function of progenitor cell-derived islet-like structures, they were transplanted under the capsule of the left kidney of diabetic mice. Blood glucose was measured regularly after transplantation. When euglycemia was maintained for one week, the left kidney with transplants was excised surgically and examined by immunohistochemistry for human C-peptide and human mitochondrion antigen as described below. Blood glucose was monitored regularly after surgery.

### Histological examination

In order to verify further the functionality of human pancreatic progenitor cell-derived beta cells *in vivo*, the animals were sacrificed at the end of experiment. Left murine kidneys were embedded in paraffin. Then murine kidneys were immunostained for human C-peptide (mouse anti-human C-peptide, Chemicon, Temecula, CA, USA) and human mitochondrion antigen (mouse anti-human mitochondrion, Millipore, Billerica, MA, USA) to confirm the survival and functionality of transplanted human islet-like structures.

### Statistical analysis

The analysis was conducted with SPSS11.0 software and the data were expressed as mean ± SD. The *t* test was uesed for comparison between two groups and one way analysis of variance (ANOVA) for comparisons among multiple groups. *P* <0.05 and P <0.01 denoted statistical significance.

## Results

### Isolation, purification and identification of pancreatic progenitor cells

**Figure 1:**
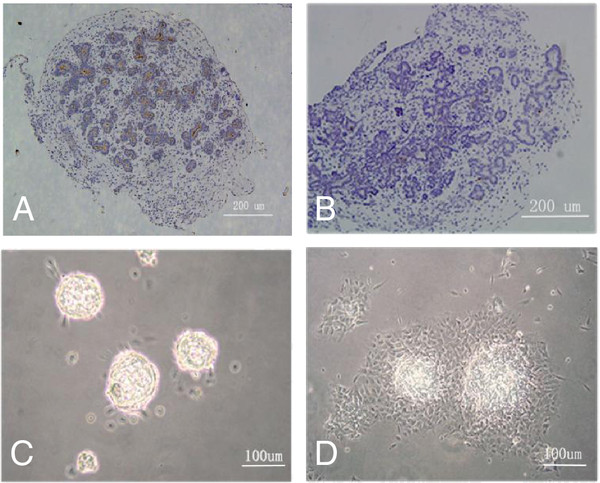
**Isolation of progenitor cells from human fetal pancreatic tissue. (A** and **B)** The histological feature of human fetal pancreas at the 11th gestational week was immunochemistry stained for CD133 **(A)** and insulin **(B)**. **(C)** Islet-like structures isolated from human fetal pancreas. (**D)** Progenitor cells grew out from the islet-like structures after 24 hours of culture. The progenitor cells exhibited epithelial-like morphology.

Between 10 to 12 weeks post conception, the pancreas contained many tubular structures within a loose mesenchymal stroma. These tubular structures consisted of epithelial cells which were CD133 positive (Figure 1A) but insulin negative (Figure 1B), indicating progenitor cells. After digestion with XI collagenase, the mesenchymal tissue was destroyed and islet-like structures were harvested (Figure 1C). The progenitor-containing clusters adhered after 24 hours and the progenitor cells began expanding (Figure 1D). These cells exhibited monolayer growth and proliferated quickly in medium containing bFGF, EGF and LIF, and confluent cells were epithelial-like (Figure 1D).

**Figure 2:**
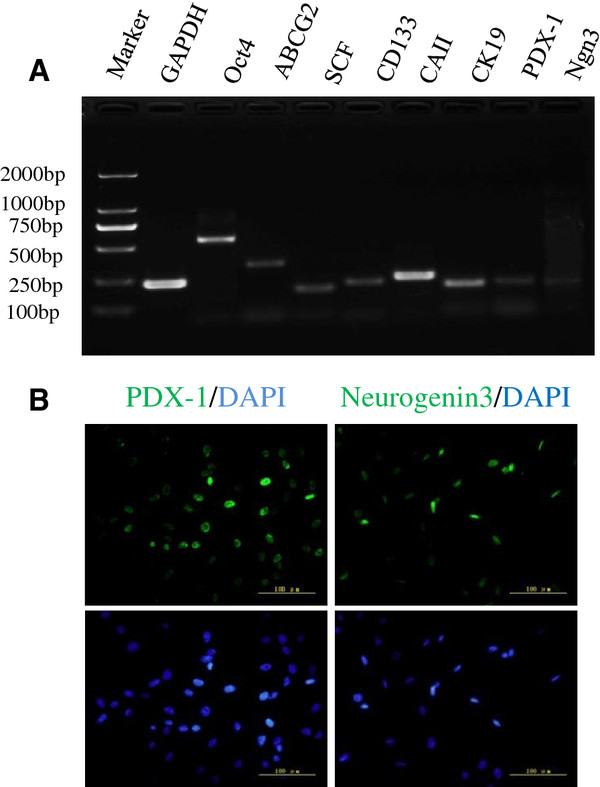
**Identification of human pancreatic progenitor cells. (A)** RT-PCR result for stem cell markers expressed on human fetal pancreatic progenitor cells. A total of five fetal samples was studied, and this image was from one sample from the 11th week of gestation. All the experiments were repeated three times. **(B)** Immunofluorescence staining for endocrine progenitor cell markers, PDX-1 and Ngn3, in human fetal pancreatic progenitor cells.

The results of RT-PCR showed that human fetal pancreatic progenitor cells expressed stem cell markers (Oct4, ABCG2, SCF and CD133), pancreatic ductal cells markers (CAII and CK19) and pancreatic endocrine markers (PDX-1 and Ngn 3) (Figure 2A). Two important stem cell markers related to pancreatic development, PDX-1 and Ngn 3, were also verified by immunofluorescence staining (Figure 2B).

To confirm the expansion of pancreatic progenitor cells rather than mesenchymal cells, the cells from the second, fifth and tenth passages were measured for expressions of SMA, vimentin and stem cells markers. The results showed that stem cell markers Oct4, CAII and PDX-1 did not fluctuate during expansion. SMA and vimentin could be detected at passage two and the expression levels were maintained during expansion (see Additional file 2) but insulin and glucagon were not detectable in these cells.

### Differentiation of pancreatic progenitor cells

To evaluate the differentiation of pancreatic progenitor cells, several important stem cell markers and mature endocrine cell markers were measured after differentiation. The results of real-time PCR (Table 1) showed that PDX-1 and neurogenin 3 were up-regulated during differentiation. Insulin and glucagon expression became detectable after a three-week induction period and were significantly enhanced at Week 4, especially in islet-like structures. GLUT1 was expressed in pancreatic stem cells and increased during differentiation. VDCC, another mature marker of β cells, could not be detected in pancreatic stem cells until week 3 of induction and became further up-regulated at Week 4, especially in islet-like structures. In addition, the exocrine markers of HES-1 and amylase were also detectable, but their expression levels did not fluctuate significantly before or after differentiation (Table 1).

**Table 1:** Quantitative analysis of various gene transcripts during differentiation

	**GAPDH**	**PDX-1**	**Ngn3**	**CAII**	**Insulin**	**Glucagon**	**GLUT1**	**GLUT2**	**VDCC**	**Hes1**	**Amylase**
0w cells	20.14	34.63	37.63	23.74	---	---	29.56	---	---	33.83	31.55
3w cells	20.78	33.80	36.80	18.87	31.23	29.40	28.80	33.87	33.86	33.46	31.41
4w cells	20.76	33.87	36.87	19.76	27.43	26.20	27.89	31.84	31.17	33.81	31.10
4w-islets	20.90	32.81	35.81	20.01	25.61	24.32	26.79	29.58	29.26	33.90	32.02

Data are means of △ cycle threshold values from cells of five different samples. W, week.

### Formation of islet-like structures in vitro

**Figure 3:**
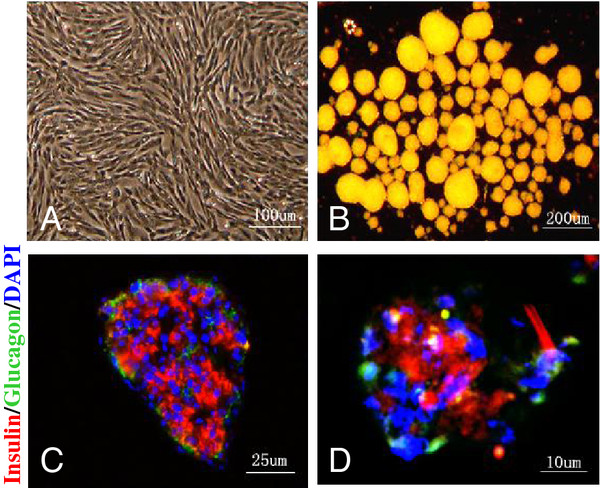
**Differentiation of pancreatic progenitor cells and formation of islet-like structures. (A)** The endocrine cells differentiated from human fetal pancreatic progenitor cells, ×200. **(B)** Islet-like structure formed by the differentiated cells, ×100. **(C)** Native islet immunofluorescence stained for insulin (red) and glucagon (green), DAPI used for nuclei staining (blue), ×400. **(D)** Progenitor cell-derived islet immunofluorescence stained for insulin (red) and glucagon (green), DAPI used for nuclei staining (blue), ×400. DAPI, 4',6-diamidino-2-phenylindole.

After four weeks of induction, the cells started exhibiting fibroblast-like morphology (Figure 3A). Upon suspension with extracellular matrix, islet-like structures formed in the culture (Figure 3B). Unlike native human islets with insulin-positive cells found in the center and glucagon-positive cells at the periphery (Figure 3C), these islet-like cell clusters had alpha and beta cells scattered about (Figure 3D).

### Insulin-releasing function of differentiated cells in vitro

**Figure 4:**
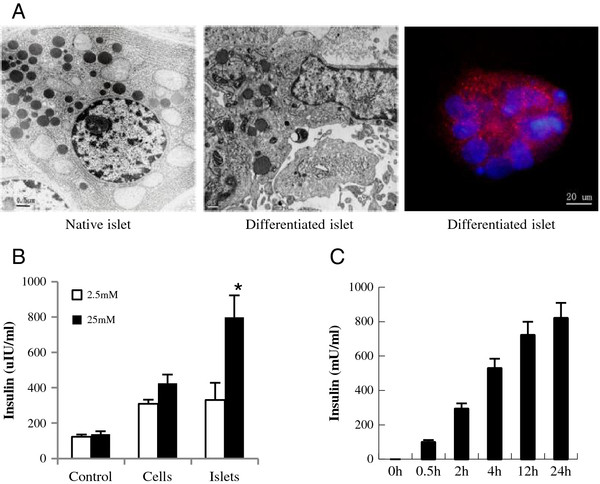
**Evaluation of insulin secretory function of differentiated cells *in vitro*. (A)** Detection of insulin secretory granules. Insulin granules in the differentiated cells were detected by transmission electron microscopy (middle panel), with adult islet beta cells as positive control (left panel); the insulin distribution in differentiated cells was granular by immunofluorescence staining (right panel). **(B)** Insulin release in differentiated cells and islet-like structures upon glucose stimulation. **(C)** The time-effect relationship of insulin release in the response of differentiated cells to glucose. All the experiments were repeated three times, and the results are presented as means ± SD.

To evaluate the insulin-releasing function of differentiated cells, we first examined the insulin secretory granules by transmission electron microscopy and immunfluorescence staining. Both experiments confirmed the existence of insulin secretory granules although much fewer than can be seen in native islets (Figure 4A). Then, insulin secretion of differentiated cells in response to glucose stimulation was tested by ELISA*.* The results showed that the response to 25 mM glucose stimulation seemed quite robust (Figure 4B) with insulin gradually increasing over 24 hours (Figure 4C).

### Functions of differentiated cells in vivo

**Figure 5:**
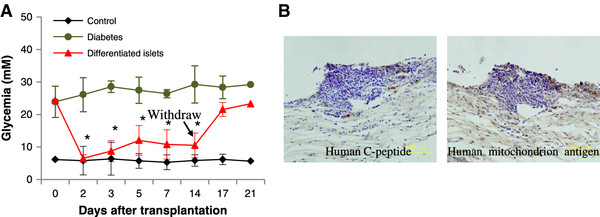
**Long-term evaluation of glycemia in diabetic animals after transplantation with islets derived from human fetal pancreas progenitor cells. (A)** Glycemia in diabetic nude mice after transplantation with progenitor cell differentiated islets. Normal mice comprise the control group. N = 6 in each group. *P <0.05 (progenitor cells group versus diabetes group). **(B)** Evaluation of the survival of grafted islets by immunohistochemistry staining for human C-peptide and human mitochondrion antigen.

To examine their function *in vivo*, we transplanted the differentiated islet-like structures under the left kidney capsule of diabetic mice. The blood glucose level in the differentiated islets group decreased significantly compared to the diabetic group two days after transplantation (*P* <0.05) and stayed below 10 mM. There was no significant difference in the blood glucose level between the differentiated islets group and the control group two days after transplantation. Hyperglycemia returned (over 20 mM) after left nephrectomy along with transplanted islet-like structures (Figure 5A). Human C-peptide-positive cells and human mitochondrion antigen-positive cells were found under the capsule of the left kidney (Figure 5B), which demonstrated the function and survival of the graft.

This evidence suggests that the islet-like structures derived from human fetal pancreatic progenitor cell can survive and function *in vivo*.

## Discussion

Pancreatic β cell dysfunction and/or loss of pancreatic β cell mass are key pathogenic factors for diabetes mellitus. Although a preferred therapeutic strategy, islet transplantation has been impeded by limited supply. Therefore, interest in generating insulin-producing cells efficiently became inevitable. Our study shows that human fetal pancreatic progenitor cells can be expanded extensively and differentiated into pancreatic endocrinal cells and islet-like structures *in vitro*. After transplantation of such structures into diabetic animals, euglycemia can be successfully restored and maintained.

It has been reported that LIF, important for self-renewal, can maintain stem cells in an undifferentiated stage [22] and bFGF plays a crucial role in cell proliferation and differentiation during embryonic development [12]. Therefore, we expanded human fetal pancreatic progenitor cells *in vitro* using culture medium containing LIF and bFGF. Our study shows that human fetal pancreatic progenitor cells exhibit a strong proliferation capability in this culture medium. The number of these cells from one single human fetal pancreas could be expanded to 1 × 10^10^.

During expansion, mesenchymal cell markers of SMA and vimentin became positive at passage two and stayed at the same level during the entire period. Due to the high expression of stem cell marker in these cells, EMT was suspected. Human pancreatic progenitors were reported to transform into mesenchymal-like cells through EMT in a growth-promoting medium. Upon reversal of EMT, these cells could form islet-like aggregates of hormone-producing cells [17,23].

Similar to adult pancreatic stem cells, human fetal pancreatic progenitor cells expressed PDX-1 and neurogenin-3. As a marker related to islet differentiation, PDX-1 was expressed during differentiation into endocrine cells [15,24]. Other studies indicated that embryonic stem cells differentiating into insulin-producing cells were also nestin positive [5]. As a poly(ADP-ribose) synthetase inhibitor, nicotinamide promotes cell differentiation and the expressions of insulin and C peptide [25]. Thus, it has been widely used in studies of stem cells differentiating into the pancreatic endocrine lineage. Glucagon-like peptide-1, all-trans retinoic acid and activin A also have been demonstrated to be effective in inducing embryonic stem cell transformation into insulin-producing cells [26]. After induction of human fetal pancreatic progenitor cells with nicotinamide, glucagon-like peptide-1, all-trans retinoic acid and activin A, the expression of mature endocrine cell markers, such as insulin, glucagon, GLUT2 and VDCC, increased extensively and became further up-regulated in islet-like structures.

It was found that islet-like structures formed when differentiated endocrine cells were cultured in suspension with medium containing extracellular matrix molecules, such as laminin, collagen IV and fibronectin. The extracellular matrix molecules mediated adherence between β cells and contributed to proliferation and differentiation into insulin-producing cell [27]. They could also enhance islets survival, insulin expression and release in response to glucose [28-30].

The insulin-releasing response to glucose (25 mM) was found in differentiated cells and it was even stronger in differentiated islet-like structures. Our previous study showed that alpha-cell loss from islets impaired insulin secretion *in vitro* and *in vivo*[31]. In the present study, immunofluorescent staining revealed that islet-like structures contained both alpha and beta cells. However, the distribution of alpha cells in the islet-like structures was different from that in native islets.

It is known that insulin-producing cells derived from human embryonic stem cells or human pancreatic islet progenitor cells could correct hyperglycemia in diabetic mice [32,33]. In the present study, differentiated islet-like structures derived from human fetal pancreatic progenitor cells could release insulin upon *in vitro* stimulation with glucose and corrected hyperglycemia in diabetic animals *in vivo*. Furthermore, histological examination revealed that the grafted islet-like structures were of human origin and immune-positive for human C-peptide.

## Conclusions

With an excellent capacity for proliferation, human fetal pancreatic progenitor cells may be induced to differentiate into insulin-producing cells and form islet-like structures *in vitro*. Capable of secreting insulin to reduce hyperglycemia after transplantation in diabetic animals, the resulting islets might become a potential source for islets transplantation in treatment for diabetes.

## Abbreviations

ABCG2: ATP-binding cassette superfamily G member 2; bFGF: Basic fibroblast growth factor; BSA: Bovine serum albumin; CAII: Carbonic anhydrase II; CK19: Cytokaretin 19; (D)MEM: (Dulbecco’s) modified Eagle’s medium; EGF: Epidermal growth factor; ELISA: Enzyme-linked immunosorbent assay; EMT: Epithelial to mesenchymal transition; ESC: Embryonic stem cells; FBS: Fetal bovine serum; GLUT: Glucose transporter; IEQ: Islet equivalent; iPS: Induced pluripotent stem; LIF: Leukemia inhibitor factor; Ngn3: Neurogenin 3; PBS: Phosphate-buffered saline; PDX-1: Pancreatic duodenal homeobox-1; RT-PCR: Real time-polymerase chain reaction; SCF: Stem cell factor; SMA: Smooth muscle actin; VDCC: Voltage-dependent calcium channel.

## Competing interests

The authors declare that they have no competing interests.

## Authors’ contributions

LJN and DHK designed the research. LH contributed to sample collection. ZWJ, CHQ, XSQ, MXL, WZ, JYW and LHL performed experiments and analyzed data. LCH and CL contributed to the research design and data analysis. SWG contributed to the evaluation of islets function *in vivo*. ZWJ and LJN were responsible for manuscript composition. All authors read and approved the final manuscript.

## Supplementary Material

Additional file 1 The gene-specific primer sequences for PCR amplification. Click here for file

Additional file 2 **The cells passaged two, five and ten times were used for real-time PCR, and the stem cell marker OCT4, ductal cell marker CAII, endocrine marker PDX1 as well as mesenchymal marker SMA and Vimentin were detected.** The experiment was repeated three times. Click here for file

## References

[B1] Noguchi H Production of pancreatic beta-cells from stem cells Curr Diabetes Rev 2010 4 184 190 10.2174/157339910791162934 20380628

[B2] Shapiro AM Lakey JR Ryan EA Korbutt GS Toth E Warnock GL Kneteman NM Rajotte RV Islet transplantation in seven patients with type 1 diabetes mellitus using a glucocorticoid-free immunosuppressive regimen N Engl J Med 2000 4 230 238 10.1056/NEJM200007273430401 10911004

[B3] Ryan EA Lakey JR Rajotte RV Korbutt GS Kin T Imes S Rabinovitch A Elliott JF Bigam D Kneteman NM Warnock GL Larsen I Shapiro AM Clinical outcomes and insulin secretion after islet transplantation with the Edmonton protocol Diabetes 2001 4 710 719 10.2337/diabetes.50.4.710 11289033

[B4] Nostro MC Keller G Generation of beta cells from human pluripotent stem cells: potential for regenerative medicine Semin Cell Dev Biol 2012 4 701 710 10.1016/j.semcdb.2012.06.010 22750147 PMC4400853

[B5] Lumelsky N Blondel O Laeng P Velasco I Ravin R McKay R Differentiation of embryonic stem cells to insulin-secreting structures similar to pancreatic islets Science 2001 4 1389 1394 10.1126/science.1058866 11326082

[B6] Assady S Maor G Amit M Itskovitz-Eldor J Skorecki KL Tzukerman M Insulin production by human embryonic stem cells Diabetes 2001 4 1691 1697 10.2337/diabetes.50.8.1691 11473026

[B7] Kunisada Y Tsubooka-Yamazoe N Shoji M Hosoya M Small molecules induce efficient differentiation into insulin-producing cells from human induced pluripotent stem cells Stem Cell Res 2012 4 274 284 10.1016/j.scr.2011.10.002 22056147

[B8] Kadam S Govindasamy V Bhonde R Generation of functional islets from human umbilical cord and placenta derived mesenchymal stem cells Methods Mol Biol 2012 4 291 313 10.1007/978-1-61779-815-3_17 22610566

[B9] Chandra V Swetha G Muthyala S Jaiswal AK Bellare JR Nair PD Bhonde RR Islet-like cell aggregates generated from human adipose tissue derived stem cells ameliorate experimental diabetes in mice PLoS One 2011 4 e20615 10.1371/journal.pone.0020615 21687731 PMC3110196

[B10] Matveyenko AV Georgia S Bhushan A Butler PC Inconsistent formation and nonfunction of insulin-positive cells from pancreatic endoderm derived from human embryonic stem cells in athymic nude rats Am J Physiol Endocrinol Metab 2010 4 E713 E720 10.1152/ajpendo.00279.2010 20587750 PMC3774125

[B11] Zulewski H Abraham EJ Gerlach MJ Daniel PB Moritz W Muller B Vallejo M Thomas MK Habener JF Multipotential nestin-positive stem cells isolated from adult pancreatic islets differentiate ex vivo into pancreatic endocrine, exocrine, and hepatic phenotypes Diabetes 2001 4 521 533 10.2337/diabetes.50.3.521 11246871

[B12] Atouf F Choi Y Fowler MJ Poffenberger G Vobecky J Ta M Chapman GB Powers AC Lumelsky NL Generation of islet-like hormone-producing cells in vitro from adult human pancreas Cell Transplant 2005 4 735 748 10.3727/000000005783982602 16454348

[B13] Li G Ye L Li J Yang W Lou J Impact of islet alpha cell loss on insulin secretion Zhonghua Yi Xue Za Zhi 2002 4 1427 1431 12509929

[B14] Ramiya VK Maraist M Arfors KE Schatz DA Peck AB Cornelius JG Reversal of insulin-dependent diabetes using islets generated in vitro from pancreatic stem cells Nat Med 2000 4 278 282 10.1038/73128 10700229

[B15] Bonner-Weir S Taneja M Weir GC Tatarkiewicz K Song KH Sharma A O’Neil JJ In vitro cultivation of human islets from expanded ductal tissue Proc Natl Acad Sci USA 2000 4 7999 8004 10.1073/pnas.97.14.7999 10884429 PMC16659

[B16] Noguchi H Naziruddin B Jackson A Shimoda M Ikemoto T Fujita Y Chujo D Takita M Kobayashi N Onaca N Hayashi S Levy MF Matsumoto S Characterization of human pancreatic progenitor cells Cell Transplant 2010 4 879 886 10.3727/096368910X509004 20587146

[B17] Joglekar MV Joglekar VM Joglekar SV Hardikar AA Human fetal pancreatic insulin-producing cells proliferate in vitro J Endocrinol 2009 4 27 36 10.1677/JOE-08-0497 19171567

[B18] Zhang L Hu J Hong TP Liu YN Wu YH Li LS Monoclonal side population progenitors isolated from human fetal pancreas Biochem Biophys Res Commun 2005 4 603 608 10.1016/j.bbrc.2005.05.111 15946651

[B19] Yao ZX Qin ML Liu JJ Chen XS Zhou DS In vitro cultivation of human fetal pancreatic ductal stem cells and their differentiation into insulin-producing cells World J Gastroenterol 2004 4 1452 1456 15133852 10.3748/wjg.v10.i10.1452 PMC4656283

[B20] Wu F Jagir M Powell JS Long-term correction of hyperglycemia in diabetic mice after implantation of cultured human cells derived from fetal pancreas Pancreas 2004 4 e23 e29 10.1097/00006676-200407000-00064 15211120

[B21] Ricordi C Lacy PE Scharp DW Automated islet isolation from human pancreas Diabetes 1989 4 140 142 2642838 10.2337/diab.38.1.s140

[B22] Zandstra PW Le HV Daley GQ Griffith LG Lauffenburger DA Leukemia inhibitory factor (LIF) concentration modulates embryonic stem cell self-renewal and differentiation independently of proliferation Biotechnol Bioeng 2000 4 607 617 10.1002/1097-0290(20000920)69:6<607::AID-BIT4>3.0.CO;2-F 10918135

[B23] Dalvi MP Umrani MR Joglekar MV Hardikar AA Human pancreatic islet progenitor cells demonstrate phenotypic plasticity in vitro J Biosci 2009 4 523 528 10.1007/s12038-009-0071-x 19920338

[B24] Kaneto H Matsuoka TA Miyatsuka T Kawamori D Katakami N Yamasaki Y Matsuhisa M PDX-1 functions as a master factor in the pancreas Front Biosci 2008 4 6406 6420 18508668 10.2741/3162

[B25] Otonkoski T Beattie GM Mally MI Ricordi C Hayek A Nicotinamide is a potent inducer of endocrine differentiation in cultured human fetal pancreatic cells J Clin Invest 1993 4 1459 1466 10.1172/JCI116723 8104197 PMC288291

[B26] Xu X Browning VL Odorico JS Activin, BMP and FGF pathways cooperate to promote endoderm and pancreatic lineage cell differentiation from human embryonic stem cells Mech Dev 2011 4 412 427 10.1016/j.mod.2011.08.001 21855631 PMC3225072

[B27] Jiang FX Cram DS DeAizpurua HJ Harrison LC Laminin-1 promotes differentiation of fetal mouse pancreatic beta-cells Diabetes 1999 4 722 730 10.2337/diabetes.48.4.722 10102687

[B28] Parnaud G Hammar E Rouiller DG Armanet M Halban PA Bosco D Blockade of beta1 integrin-laminin-5 interaction affects spreading and insulin secretion of rat beta-cells attached on extracellular matrix Diabetes 2006 4 1413 1420 10.2337/db05-1388 16644699

[B29] Kaido T Yebra M Cirulli V Montgomery AM Regulation of human beta-cell adhesion, motility, and insulin secretion by collagen IV and its receptor alpha1beta1 J Biol Chem 2004 4 53762 53769 10.1074/jbc.M411202200 15485856

[B30] Wang RN Rosenberg L Maintenance of beta-cell function and survival following islet isolation requires re-establishment of the islet-matrix relationship J Endocrinol 1999 4 181 190 10.1677/joe.0.1630181 10556766

[B31] Wang H Zhang W Cai H Xu S Sui W Jiang Y Deng S Lou J alpha-Cell loss from islet impairs its insulin secretion in vitro and in vivo Islets 2011 4 58 65 10.4161/isl.3.2.15036 21372634

[B32] Kroon E Martinson LA Kadoya K Bang AG Kelly OG Eliazer S Young H Richardson M Smart NG Cunningham J Agulnick AD D’Amour KA Carpenter MK Baetge EE Pancreatic endoderm derived from human embryonic stem cells generates glucose-responsive insulin-secreting cells in vivo Nat Biotechnol 2008 4 443 452 10.1038/nbt1393 18288110

[B33] Zhang Y Ren Z Zou C Wang S Luo B Li F Liu S Zhang YA Insulin-producing cells from human pancreatic islet-derived progenitor cells following transplantation in mice Cell Biol Int 2011 4 483 490 10.1042/CBI20100152 21080910

