# Supplementary material for: Evaluation of islets derived from human fetal pancreatic progenitor cells in diabetes treatment
Source: Stem Cell Res Ther. 2013 Nov 22;4(6):141. doi: 10.1186/scrt352 (PMC4055010; doi:10.1186/scrt352)
Supplement: Additional file 1 — The gene-specific primer sequences for PCR amplification. [file scrt352-S1.doc]

| Human Genes | Primer sequences | Length of product(bp) |
| --- | --- | --- |
| GAPDH | CTGCACCACCAACTGCTTAG  GAGCTTCCCGTTCAGCTCAG | 232 |
| Oct4 | CGACCATCTGCCGCTTTGAG  CCCCCTGTCCCCCATTCCTA | 573 |
| ABCG2 | GGCCTCAGGAAGACTTATGT  AAGGAGGTGGTGTAGCTGAT | 342 |
| SCF | CTCCTATTTAATCCTCTCGTC  TACTACCATCTCGCTTATCCA | 177 |
| CD133 | CCTTGTGGCAAAGCTCAACC  TCACCTCCTCTCTCACCCAG | 215 |
| carbonic anhydrase II(CA II) | CTGGGGTTCACTTGATGGACA  GAAGTCAGCACTCTTGCCCT | 238 |
| CK19 | ACCAAGTTTGAGACGGAACAG  CCCTCAGCGTACTGATTTCCT | 181 |
| PDX-1 | ATGAAGTCTACCAAAGCTCACG  TGATGTGTCTCTCGGTCAAGTT | 208 |
| Neurogenin 3 | TTCTTTTCTCCTTTGGGGCTGG  ACGGGTCACTTGGACAGTGG | 184 |
| Insulin | CTCACACCTGGTGGAAGCTC  AGAGGGAGCAGATGCTGGTA | 212 |
| Glucagon | GATAATCTTGCCGCCAGGGA  TGGCATGCAAAGCAATGTGG | 185 |
| GLUT1 | GGCCAAGAGTGTGCTAAAGAA  ACAGCGTTGATGCCAGACAG | 201 |
| GLUT2 | TGTGGCAGCTGCTCAACTAA  AGGCCTGAAATTAGCCCACAA | 255 |
| VDCC | GAAGCGGCAGCAATATGGGA  TTGGTGGCGTTGGAATCATCT | 225 |
| HES-1 | TGAAAGTCTGAGCCAGCTGAA  GTACTTCCCCAGCACACTTG | 178 |
| Amylase | AGTGGATGGCCAGCCTTTTA  GAGCTTTGCCATCGTCAGAA | 227 |
| SMA | CTATGAGGGCTATGCCTTGCC  GCTCAGCAGTAGTAACGAAGGA | 122 |
| Vimentin | GACGCCATCAACACCGAGTT  CTTTGTCGTTGGTTAGCTGGT | 238 |

**Additional file 1:**

**The gene-specific primers for PCR.**
