# Supplementary figures and images for: Evaluation of islets derived from human fetal pancreatic progenitor cells in diabetes treatment
Source: Stem Cell Res Ther. 2013 Nov 22;4(6):141. doi: 10.1186/scrt352 (PMC4055010; doi:10.1186/scrt352)

## Slide 1
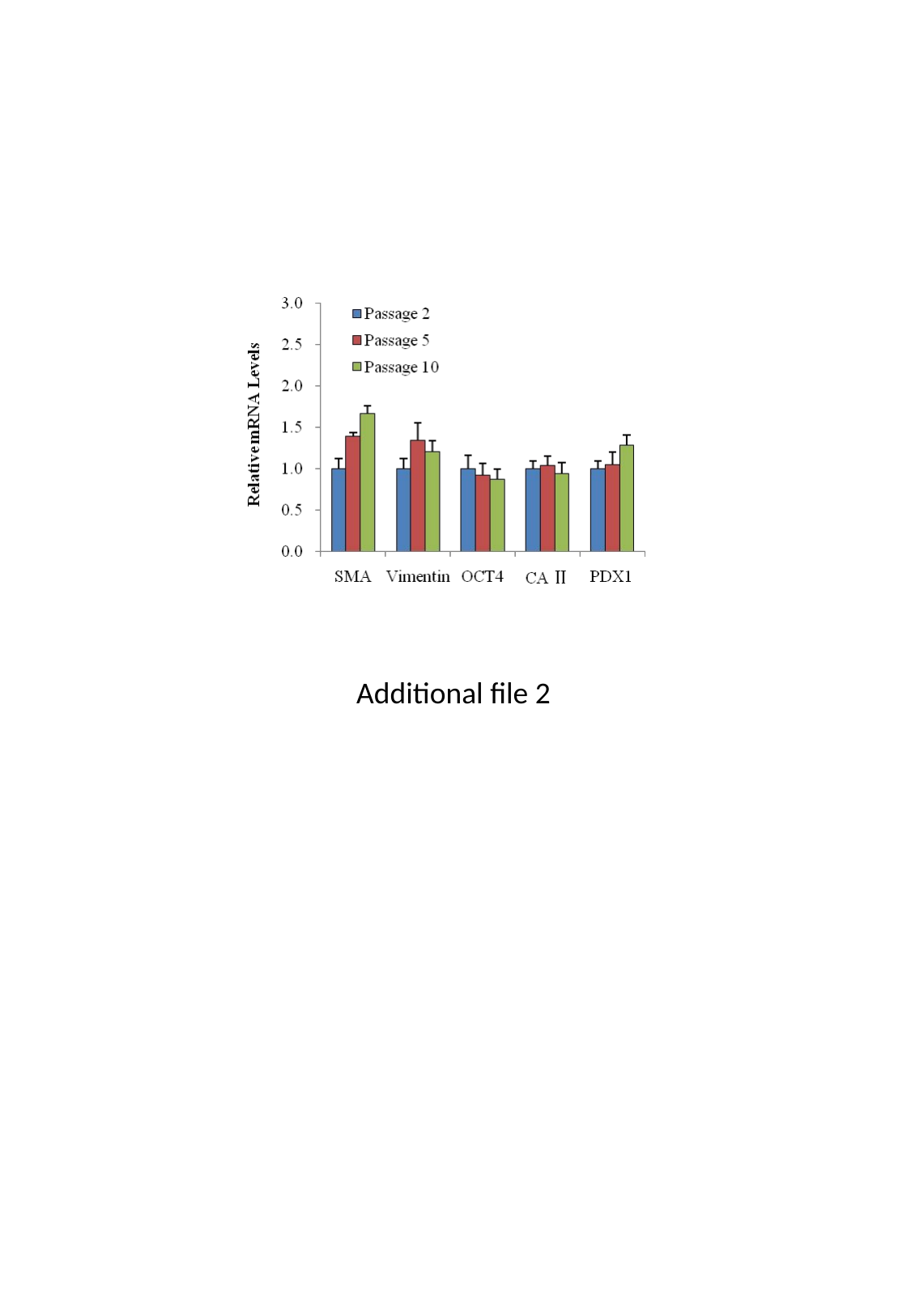

Additional file 2

Supplement: Additional file 2 — The cells passaged two, five and ten times were used for real-time PCR, and the stem cell marker OCT4, ductal cell marker CAII, endocrine marker PDX1 as well as mesenchymal marker SMA and Vimentin were detected. The experiment was repeated three times. [file scrt352-S2.ppt]
